# Time-varying intensity of oxygen exposure is associated with mortality in critically ill patients with mechanical ventilation

**DOI:** 10.1186/s13054-022-04114-w

**Published:** 2022-08-05

**Authors:** Zhu Zhu, Mingqin Zhou, Yao Wei, Hui Chen

**Affiliations:** 1 Department of General Surgery, Suzhou Science & Technology Town Hospital, Suzhou, 215153 Jiangsu People’s Republic of China; 2 Department of Critical Care Medicine, Cancer Hospital of Shantou University Medical College, No.7 Raoping Road, Shantou, 515100 Guangdong People’s Republic of China; 3 Department of Critical Care Medicine, The First Affiliated Hospital of Soochow University, Soochow University, No. 899 Pinghai Road, Suzhou, 215000 People’s Republic of China

**Keywords:** Oxygen exposure, Intensive care unit, Mechanical ventilation, 28-day mortality

## Abstract

**Background:**

There is no consensus exists regarding the association between oxygen exposure (arterial oxygen tension or fraction of inspired oxygen) and outcomes for patients with mechanical ventilation. Additionally, whether the association remains persistent over time is unknown. We aimed to explore the association between exposure to different intensities of oxygen exposure over time and 28-day mortality in patients with mechanical ventilation.

**Methods:**

We obtained data from the Medical Information Mart for Intensive Care IV (MIMIC-IV), which included adult (≥ 18 years) patients who received invasive mechanical ventilation for at least 48 h. We excluded patients who received extracorporeal membrane oxygenation (ECMO) or who initiated ventilation more than 24 h after ICU admission. The primary outcome was 28-day mortality. Piece-wise exponential additive mixed models were employed to estimate the strength of associations over time.

**Results:**

A total of 7784 patients were included in the final analysis. Patients had a median duration of invasive mechanical ventilation of 8.1 days (IQR: 3.8–28 days), and the overall 28-day mortality rate was 26.3%. After adjustment for baseline and time-dependent confounders, both daily time-weighted average (TWA) arterial oxygen tension (PaO_2_) and fraction of inspired oxygen (FiO_2_) were associated with increased 28-day mortality, and the strength of the association manifested predominantly in the early-middle course of illness. A significant increase in the hazard of death was found to be associated with daily exposure to TWA-PaO_2_ ≥ 120 mmHg (Hazard ratio 1.166, 95% CI 1.059–1.284) or TWA-FiO_2_ ≥ 0.5 (Hazard ratio 1.496, 95% CI 1.363–1.641) during the entire course. A cumulative effect of harmful exposure (TWA-PaO_2_ ≥ 120 mmHg or TWA-FiO_2_ ≥ 0.5) was also observed.

**Conclusion:**

PaO_2_ and FiO_2_ should be carefully monitored in patients with mechanical ventilation, especially during the early-middle course after ICU admission. Cumulative exposure to higher intensities of oxygen exposure was associated with an increased risk of death.

**Supplementary Information:**

The online version contains supplementary material available at 10.1186/s13054-022-04114-w.

## Introduction

The administration of supplemental oxygen is a standard treatment in mechanically ventilated patients in Intensive Care Units (ICU). Oxygen therapy can reverse tissue hypoxia and can be lifesaving on many occasions, while its overzealous use might result in hyperoxemia with supraphysiological levels of the arterial oxygen tension (PaO_2_) [1]. Additionally, most patients with mechanical ventilation frequently required a higher fraction of inspired oxygen (FiO_2_) to maintain an adequate PaO_2_.

PaO_2_ and FiO_2_ outside the normal physiological range can be detrimental and exacerbate systemic organ injury in critically ill patients [2–5], while numerous studies provided conflicting evidence concerning the impact of PaO_2_ and FiO_2_ on clinical outcomes of critically ill patients [6–10]. The cut-off values of PaO_2_ and FiO_2_ associated with increased risk of death were various, and still uncertain. Meanwhile, previous studies focused on only the association between PaO_2_ or FiO_2_ during the early onset of illness and mortality, usually within 24 h after ICU admission, and many of these studies are limited by using only a single measure of PaO_2_ or FiO_2_ to define oxygen exposure for entire ICU admission. Whether the association between time-varying oxygen exposure and mortality is significant and remains persistent over time is unknown. Besides, high FiO_2_ can increase PaO_2_ and impair lung tissues simultaneously [11, 12], while the direct and indirect effects (mediated by hyperoxemia) of high FiO_2_ on mortality in the real world have never been explored.

Therefore, our primary objective was to estimate the effect of time-varying exposure to different intensities of oxygen exposure (as measured either by PaO_2_ or FiO_2_) on 28-day mortality in patients with mechanical ventilation. We also examined whether the strength of the effect changed over time, and whether there was a cumulative effect of exposure over time. Finally, we aimed to identify the direct and indirect effects of high FiO_2_ on mortality.

## Methods

### Study design and participants

We conducted a retrospective cohort study using electronic health records data from the Medical Information Mart for Intensive Care IV (MIMIC-IV) [13]. The MIMIC-IV database contains comprehensive and high-quality data of well-defined and characterized ICU patients admitted to ICUs at the Beth Israel Deaconess Medical Center between 2008 and 2019. One author (HC) obtained access to the database and was responsible for data extraction (certification number 27252652). Our study complied with the Reporting of Studies Conducted using Observational Routinely Collected Health Data (RECORD) statement.

All patients in the MIMIC-IV who received invasive mechanical ventilation were eligible for inclusion in the present study. The exclusion criteria included (1) Patients who were younger than 18 years; (2) Patients ventilated for less than 48 h; (3) Patients who received extracorporeal membrane oxygenation (ECMO); (4) Patients who initiated ventilation more than 24 h after ICU admission. Additionally, we analyzed only the first ICU stay for patients who were admitted to the ICU more than once.

### Variable extraction

We collected age, sex, weight, height, ethnicity, admission type, and severity at admission, as measured by the Sequential Organ Failure Assessment (SOFA) score, the Simplified Acute Physiology Score II (SAPS II), and Oxford Acute Severity of Illness Score (OASIS). Comorbidities were also collected to calculate the Elixhauser comorbidity score. Initial diagnoses were extracted according to the recorded ICD-9 and ICU-10 codes in the database. Longitudinal data including SOFA score, blood gas, ventilation parameters, and laboratory measurements were collected for each 24 h. If a variable was recorded more than once in the 24 h, we used the value related to the greatest severity of illness. Specifically, PaO_2_ and FiO_2_ were extracted per each time frame of 4 h after ICU admission. For each 24 h, the time-weighted average (TWA) PaO_2_ or FiO_2_ was calculated as the area under the PaO_2_ or FiO_2_ versus the time plot. All included patients were followed up from inclusion until death, ICU discharge, or 28 days in the ICU, whichever occurred first.

### Exposures and outcomes

The primary exposures in the present study were time-varying TWA-PaO_2_ and TWA-FiO_2_. Since our research indicated that there is a consistent increase in the risk of death with TWA-PaO_2_ higher than or equal to 120 mmHg and TWA-FiO_2_ higher than or equal to 0.5, we defined hyperoxemia and high FiO_2_ as TWA-PaO_2_ ≥ 120 mmHg, and TWA-FiO_2_ ≥ 0.5, respectively, and employed three approaches to quantifying the effect of harmful exposure: (1) Any exposure to hyperoxemia or high FiO_2_ during the entire course of ICU admission; (2) The proportion of time spent in hyperoxemia or high FiO_2_; (3) The area under the longitudinal profiles for either TWA-PaO_2_ of 120 mmHg or more, or TWA-FiO_2_ of 0.5 or more.

The primary outcome was 28-day mortality. Secondary outcomes included in-hospital mortality, ventilation-free days (VFDs) in 28 days, length of ICU, and hospital stay. Patients who died before day 28 were considered to have zero VFDs.

### Statistical analysis

Values are presented as the mean (standard deviation) or median [interquartile range (IQR)] for continuous variables as appropriate and as the total number (percentage) for categorical variables. Comparisons between groups were made using the X^2^ test or Fisher’s exact test for categorical variables and Student’s t test or Mann–Whitney U test for continuous variables as appropriate.

We first used piece-wise exponential additive mixed models (PAMMs) [14–16] to estimate the association of subject-specific longitudinal profiles of either TWA-PaO_2_ or TWA-FiO_2_ with 28-day mortality. PAMMs allow one to examine the time-varying effect of a time-varying exposure (time-varying TWA-PaO_2_ or TWA-FiO_2_ in the present study) on a time-to-event outcome (Additional file 1). Based on prior knowledge, baseline variables were purposefully selected to be used in the PAMMs as time-fixed confounders and included age, gender, admission type, weight, and Elixhauser comorbidity score. Considering that the confounders may change over time during the follow-up period, and that the effect of such confounders on outcomes is time-varying, we treated longitudinal data including the use of mechanical ventilation and vasopressor, SOFA score, and PaCO_2_ during follow-up as time-varying confounders in the PAMMs. To investigate whether the association between TWA-PaO_2_ or TWA-FiO_2_ and 28-day mortality changed over time, we included an interaction term with time and exposures in the model. To avoid bias induced by missing data, we used multiple imputations by chained equation (MICE) to account for the missing data.

We identified the approximate PaO_2_ and FiO_2_ threshold above which the risk of 28-day mortality began to increase based on PAMMs. And then, three secondary analyses were performed. We included any hyperoxemia or high FiO_2_ as time-varying exposure variables and explored the impact of any exposure to hyperoxemia or high FiO_2_ on mortality; we also estimated the association between the proportion of time spent in harmful exposure (TWA-PaO_2_ ≥ 120 mmHg or TWA-FiO_2_ ≥ 0.5) and mortality. Furthermore, we investigated the relationship between cumulative dose and 28-day mortality using the area under the longitudinal profiles for either TWA-PaO_2_ of 120 mmHg or more, or TWA-FiO_2_ of 0.5 or more.

Based on the additive hazards model, we conduct a causal mediation analysis (CMA) to explore whether the effect of time-varying high FiO_2_ (TWA-FiO_2_ ≥ 0.5) on the primary outcome is mediated by the time-varying hyperoxemia. The additive hazards model allows for a time-varying mediator in survival analysis [17]. CMA separates the total effect of exposure into direct and indirect effects. The indirect impact on the outcome is mediated via a mediator. Our study used time-varying high FiO_2_ as a harmful exposure and time-varying hyperoxemia as a mediator variable.

Several subgroup analyses were performed according to exposure window (up to 3 days, 5 days, 7 days, 14 days, and 21 days), admission type, gender, and initial diagnosis. Since patients could liberate from mechanical ventilation during the follow-up period, we duplicated the analysis after changing the follow-up period. Specifically, patients were followed up from inclusion until death, liberated from mechanical ventilation, ICU discharge, or 28 days in the ICU, whichever occurred first. We also conducted sensitivity analyses after excluding missing values of daily TWA-PaO_2_ or TWA-FiO_2_ during follow-up.

All statistical analyses were performed using R (version 4.0.3), and *p* < 0.05 was considered statistically significant.

## Results

### Patients in study

After reviewing the data of 29,119 patients with mechanical ventilation, a total of 7784 patients were included in the final analysis. The flow diagram of patient selections is presented in Additional file 2: Figure S1. Baseline characteristics are shown in Table 1. The patients had a median age of 65.0 years (IQR: 54.0–76.0), and 57.2% of them were men. Of the study cohort, 89.5% of patients had sepsis and 50.6% had acute respiratory failure. Patients had a median duration of invasive mechanical ventilation of 8.1 days (IQR: 3.8–28) and the overall 28-day mortality rate was 26.3%.

**Table 1:** Baseline characteristics and outcomes in included patients

	All patients(*n* = 7784)	Missing, *n* (%)
Age (years)	65.0 (54.0–76.0)	0 (%)
Male (gender)	4452 (57.2)	0 (%)
Weight (kg)	80.0 (66.7–97.0)	202 (2.6%)
Height (cm)	170.0 (163.0–178.0)	1384 (17.8%)
*Admission type, n (%)*		0 (%)
Medical	4714 (60.6)	
Surgical elective	153 (2.0)	
Surgical urgent	1830 (23.5)	
Other	1087 (14.0)	
*Ethnicity, n (%)*		0 (%)
White	4695 (60.3)	
Black	823 (10.6)	
Hispanic	266 (3.4)	
Other	1787 (23.0)	
Elixhauser comorbidity score	6.0 (4.0–8.0)	0 (%)
*Severity of illness*		
GCS	14.6 (1.1)	4 (0.1%)
SAPS II	43.0 (33.0–54.0)	0 (%)
OASIS	42.0 (36.0–47.2)	0 (%)
SOFA	3.0 (2.0–5.0)	820 (10.5%)
Use of vasopressor (1 st 24 h)	4462 (57.3)	0 (%)
*Initial diagnosis, n (%)*		0 (%)
Sepsis	6964 (89.5)	
Acute respiratory failure	3938 (50.6)	
Cardiac arrest	493 (6.3)	
*Vital signs (1 st 24 h)*		
Heart rate (bpm)	86.5 (75.2–99.1)	1 (0.0%)
MAP (mmHg)	75.8 (70.4–82.6)	1 (0.0%)
Temperature (℃)	37.0 (36.7–37.4)	463 (5.9%)
*Parameters of mechanical ventilation (1 st 24 h)*		
TWA-FiO_2_	0.50 (0.42–0.59)	6 (0.1%)
Total respiratory rate (bpm)	20.1 (17.5–23.0)	12 (0.2%)
Tidal volume (ml/kg PBW)	7.0 (6.4–7.8)	1385 (17.8%)
PEEP (cmH_2_O)	6.1 (5.1–8.1)	2 (0.0%)
Plateau pressure (cmH_2_O)	18.8 (16.1–22.0)	297 (3.8%)
Use of NMBA, *n* (%)	413 (5.3)	0 (%)
*Laboratory data (1 st 24 h)*		
pH	7.4 (7.3–7.4)	1221 (15.7%)
PaCO_2_ (mmHg)	39.8 (35.0–45.0)	1221 (15.7%)
TWA-PaO_2_ (mmHg)	130.5 (100.5–171.0)	1240 (15.9%)
Lactate (mmol/L)	2.0 (1.3–3.3)	1829 (23.5%)
Bicarbonate (mmol/L)	22.2 (19.5–25.3)	46 (0.6%)
Fluid balance in the first 24 h (L)	1.54 (− 0.24–4.59)	90 (1.2%)
*Clinical outcomes*		
28-day mortality, n (%)	2044 (26.3)	0 (%)
In-hospital mortality, n (%)	2154 (27.7)	0 (%)
Alive and VFDs (days)	19.9 (0.0–24.2)	0 (%)
Length of ICU stay (days)	7.8 (4.9–13.1)	0 (%)
Length of hospital stay (days)	14.0 (8.0–22.0)	0 (%)

*GCS* Glasgow Coma Scale; SAPS II: Simplified acute physiology score II; *OASIS* Oxford Acute Severity of Illness Score; *SOFA* sequential organ failure assessment; *MAP* mean arterial pressure; *TWA* Time-weighted average; *FiO*_*2*_ Fraction of inspired oxygen; *PBW* Predicted body weight; *PEEP* Positive end-expiratory pressure; *NMBA* neuromuscular blocking agent; *PCO*_*2*_ partial pressure of carbon dioxide; *PO*_*2*_ partial pressure of oxygen; *VFDs* Ventilation-free days; *ICU* Intensive Care Unit

A total of 6544 (84.1%) patients had complete data of TWA-PaO_2_ on Day 1, and 58.1% of patients were exposed to hyperoxemia (TWA-PaO_2_ ≥ 120 mmHg), while 7778 (99.9%) patients had complete data of TWA-FiO_2_ on Day 1, and 52.7% of patients were exposed to TWA-FiO_2_ ≥ 0.5. There were lots of differences in patient characteristics and outcomes based on their level of TWA-PaO_2_ (Additional file 2: Table S1) and TWA-FiO_2_ (Additional file 2: Table S2) on Day 1.

### Primary analysis

**Fig. 1:**
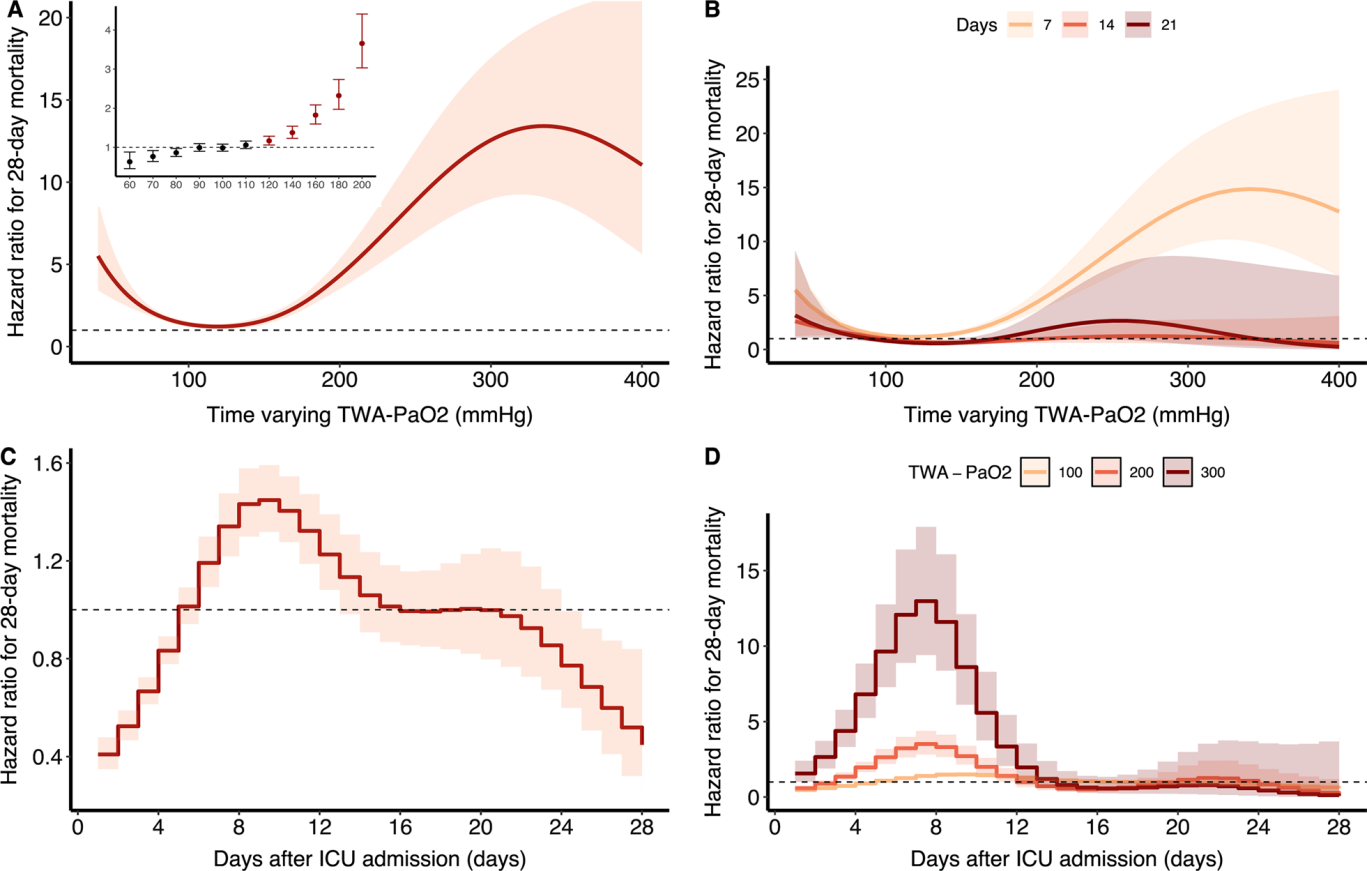
Association between daily TWA-PaO_2_ and 28-day mortality over time using piece-wise exponential additive mixed models. **A** Adjusted relationship between TWA-PaO_2_ over time and 28-day mortality; **B** Adjusted relationship between TWA-PaO_2_ over time and 28-day mortality stratified by exposure window; **C** Time-varying effect of TWA-PaO_2_ on 28-day mortality; **D** Time-varying effect of TWA-PaO_2_ on 28-day mortality stratified by level of PaO_2._ TWA: Time-weighted average; PaO_2_: Arterial oxygen tension

**Fig. 2:**
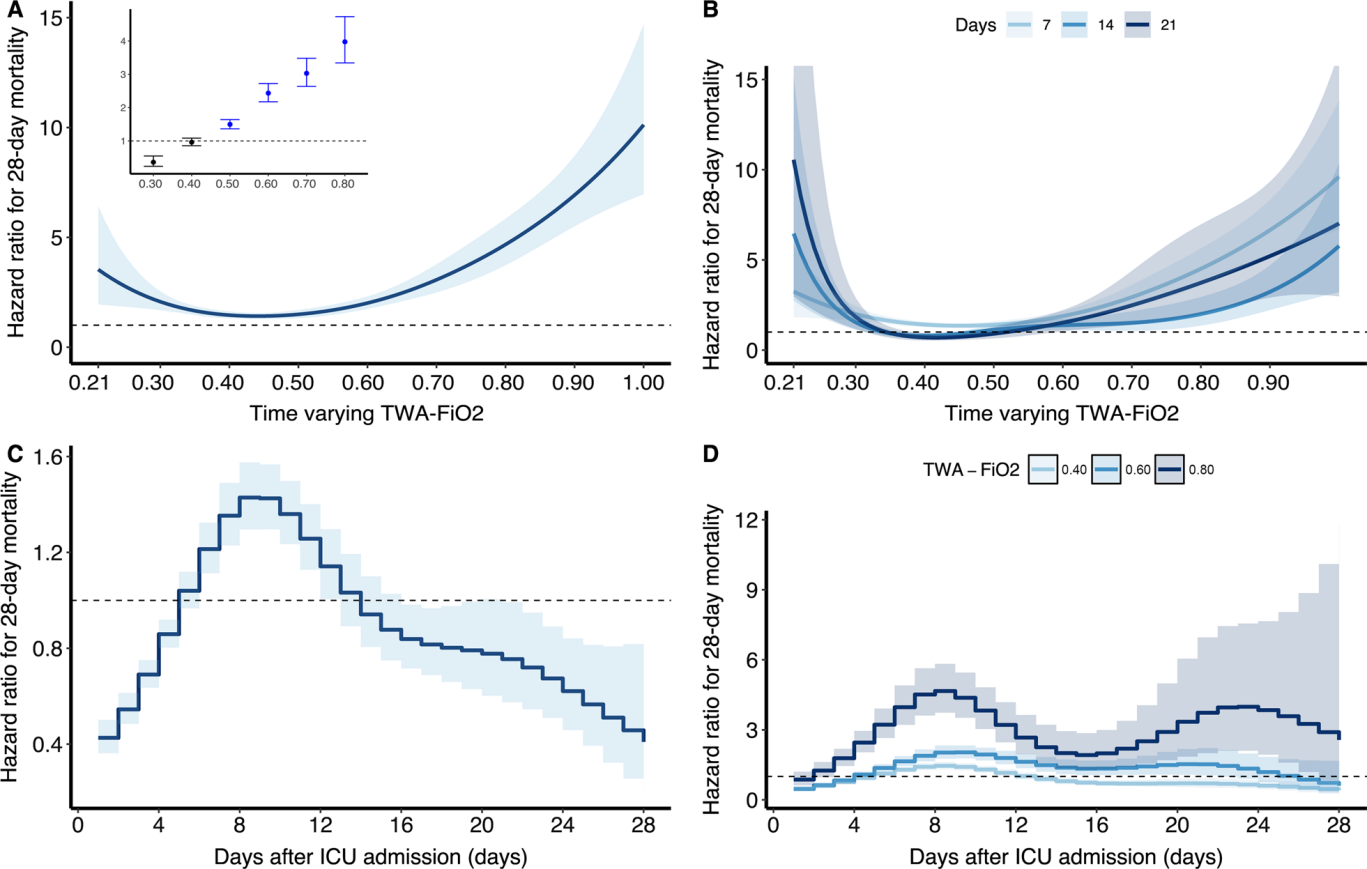
Association between daily TWA-FiO_2_ and 28-day mortality over time using piece-wise exponential additive mixed models. **A** Adjusted relationship between TWA-FiO_2_ over time and 28-day mortality; **B** Adjusted relationship between TWA-FiO_2_ over time and 28-day mortality stratified by exposure window; **C** Time-varying effect of TWA-FiO_2_ on 28-day mortality; **D** Time-varying effect of TWA-FiO_2_ on 28-day mortality stratified by level of FiO_2_. TWA: Time-weighted average; FiO_2_: Fraction of inspired oxygen

Based on the PAMMs, we identified a U-shaped relationship between time-varying TWA-PaO_2_ or TWA-FiO_2_ (Figs. 1 and 2) and the risk of 28-day mortality. There was a consistent increase in the risk of death with TWA-PaO_2_ higher than or equal to 120 mmHg and TWA-FiO_2_ higher than or equal to 0.5 (Figs. 1A and 2A). After adjusting for age, gender, admission type, weight, Elixhauser comorbidity score, use of mechanical ventilation, use of vasopressor, SOFA score, and PaCO_2_, both the time-varying TWA-PaO_2_ (Hazard ratio (HR) per 5 mmHg 1.801, 95% CI 1.585–2.046) and TWA-FiO_2_ (HR per 0.05 2.688, 95% CI 2.407–3.002) were associated with an increased risk of 28-day mortality (Additional file 2: Table S3).

The strength of the association between the intensity of oxygen exposure (as measured by either TWA-PaO_2_ or TWA-FiO_2_) and 28-day mortality was not persistent across the entire course of ICU admission (Additional file 2: Figure S2). TWA-PaO_2_ significantly impacts mortality in the early-middle course of illness (approximately 4–12 days after ICU admission) (Fig. 1C), regardless of the level of TWA-PaO_2_ (Fig. 1D). While the impacts of TWA-FiO_2_ predominantly in the early-middle course of illness (approximately 4–16 days after ICU admission) (Fig. 2C), and for the high level of TWA-FiO_2_, the impact on mortality remained persistent across the entire course of ICU admission (Fig. 2D).

### Secondary analysis

**Fig. 3:**
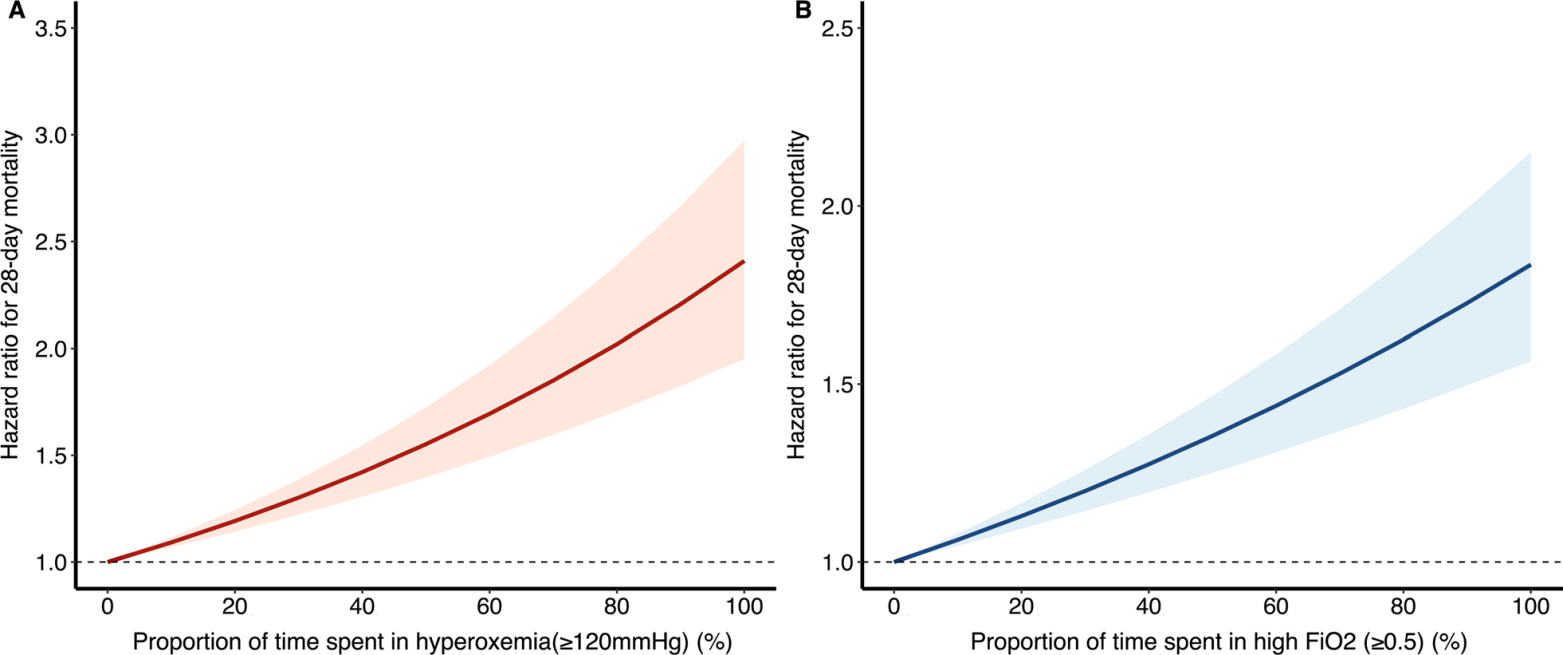
Association between proportion of time spent in hyperoxemia **A** or high FiO_2_ **B** and 28-day mortality using piece-wise exponential additive mixed models

Three secondary analyses were performed after we identified the approximate PaO_2_ and FiO_2_ threshold above which the risk of 28-day mortality began to increase. First, PAMMs demonstrated that both time-varying hyperoxemia (HR 1.166, 95% CI 1.059–1.284) and high FiO_2_ (HR 1.496, 95% CI 1.363–1.641) were associated with an increased risk of 28-day mortality (Table 2). Likewise, both the proportion of time spent in hyperoxemia (HR per 5% 1.412 95% CI 1.302–1.531) and high FiO_2_ (HR per 5% 1.284, 95% CI 1.204–1.369) significantly impact mortality (Additional file 2: Table S4 and Fig. 3). Third, a higher cumulative dose of the potentially injurious intensity of oxygen exposure was associated with an increased hazard of death for hyperoxemia (HR 1.0014, 95% CI 1.001–1.0017) and high FiO_2_ (HR 1.003, 95% CI 1·0023–1.0036) (Additional file 2: Table S5).

**Table 2:** Effect of time-varying hyperoxemia and high FiO_2_ on 28-day mortality of 7784 patients with mechanical ventilation using PAMMs

	Exposure to hyperoxemia		Exposure to high FiO_2_	
	HR (95% CI)	*P* value	HR (95% CI)	*P* value
*Baseline variables*				
Age, years	1.018(1.014–1.022)	< 0.001	1.018(1.014–1.022)	< 0.001
Male	0.981 (0.892–1.078)	0.69	0.972 (0.885–1.069)	0.57
Admission type				
Medical	Reference		Reference	
Surgical elective	0.248 (0.158–0.389	< 0.001	0.246 (0.157–0.386)	< 0.001
Surgical urgent	0.846 (0.759–0.943)	0.024	0.834 (0.748–0.929)	< 0.001
Other	0.643 (0.558–0.742)	< 0.001	0.642 (0.557–0.741)	< 0.001
Weight, kg	0.993 (0.990–0.994)	< 0.001	0.992 (0.990–0.994)	< 0.001
Elixhauser comorbidity score	1.033 (1.014–1.052)	< 0.001	1.038 (1.019–1.057)	< 0.001
*Time-varying variables*				
Receiving IMV	0.417 (0.375–0.463)	< 0.001	0.437 (0.393–0.486)	< 0.001
Use of vasopressor	1.370 (1.223–1.534)	< 0.001	1.380 (1.232–1.546)	< 0.001
SOFA score	1.145 (1.129–1.160)	< 0.001	1.131 (1.116–1.147)	< 0.001
PaCO_2_, mmHg	1.016 (1.013–1.020)	< 0.001	1.014 (1.010–1.017)	< 0.001
Any hyperoxemia (TWA-PaO_2_ ≥ 120 mmHg)	1.166 (1.059–1.284)	0.0017	–	–
Any exposure to high FiO_2_ (TWA-FiO_2_ ≥ 0.5)	–	–	1.496 (1.363–1.641	< 0.001

*PAMMs* Piece-wise exponential additive mixed models; *HR* Hazard ratio; *CI* Confidence interval; *IMV* Invasive mechanical ventilation; *SOFA* sequential organ failure assessment; *TWA* Time-weighted average; *PCO*_*2*_ partial pressure of carbon dioxide; *FiO*_*2*_ Fraction of inspired oxygen

### Causal mediation analysis

**Fig. 4:**
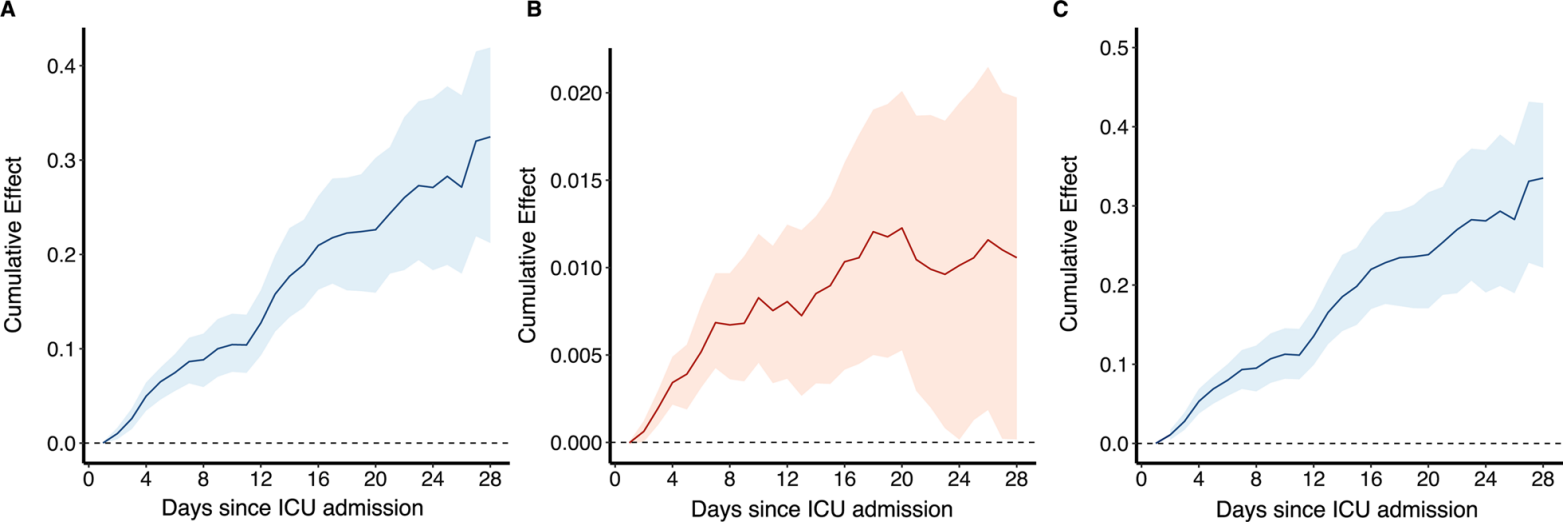
The direct effect and indirect effect of high FiO_2_ on 28-day mortality over time using the additive hazards model. **A** Direct effect of high FiO_2_ on 28-day mortality over time; **B** Indirect effect (mediated by hyperoxemia) of high FiO_2_ on 28-day mortality over time; **C** Total effect of high FiO_2_ on 28-day mortality over time

Any exposure to hyperoxemia (TWA-PaO_2_ ≥ 120 mmHg) was significantly associated with the 28-day mortality in patients with TWA-FiO_2_ ≥ 0.5 (HR 1.203, 95% CI 1.088–1.331), but not in patients with TWA-FiO_2_ < 0.5 (HR 1.056, 95% CI 0.760–1.469). Meanwhile, the association between any exposure to TWA-FiO_2_ ≥ 0.5 and 28-day mortality was significant both in patients with hyperoxemia (HR 1.60, 95% CI 1.40–1.874) and non-hyperoxemia (HR 1.556, 95% CI 1.389–1.742). To explore whether the effect of time-varying high FiO_2_ (TWA-FiO_2_ ≥ 0.5) on the primary outcome was mediated by the time-varying hyperoxemia. We treated time-varying high FiO_2_ as a harmful exposure and time-varying hyperoxemia as a mediator variable, and visual inspection showed that both the direct effect of high FiO_2_ (*p* < 0.001) and an indirect effect via hyperoxemia (*p* < 0.001) on mortality were significant (Fig. 4).

### Subgroup analyses and sensitivity analyses

**Fig. 5:**
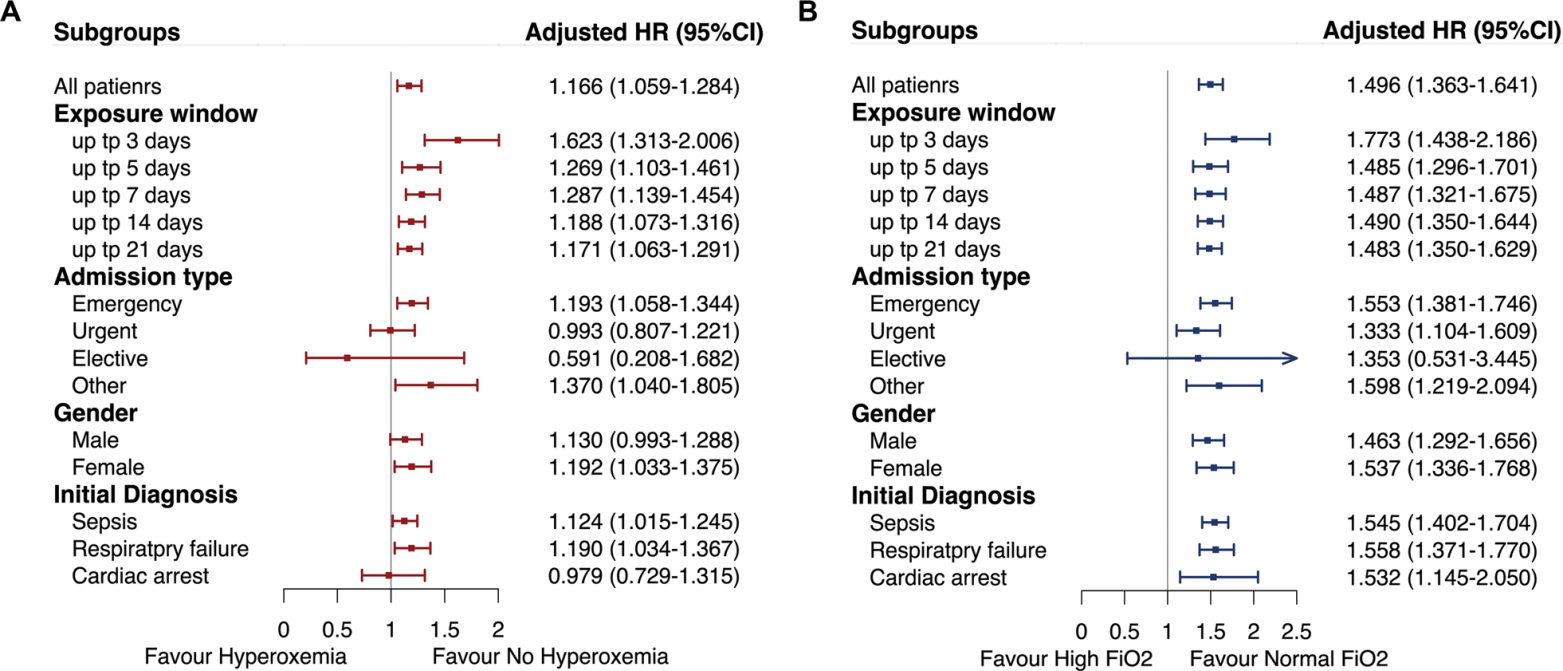
Subgroup analyses of the association between any exposure to hyperoxemia **A** or high FiO_2_ **B** and 28-day mortality over time using piece-wise exponential additive mixed models. HR = hazard ratio; CI = confidence interval

Subgroup analyses of the association between time-varying hyperoxemia and high FiO_2_ with 28-day mortality are shown in Fig. 5. Accomplished by the increase in exposure window, the hazard of death for hyperoxemia was decreased, while the hazard of death for high FiO_2_ was persistent. Two sensitivity analyses were performed. First, after excluding missing values of daily TWA-PaO_2_ or TWA-FiO_2_, any exposure to hyperoxemia (HR 1.232, 95% CI 1.059–1.435) or high FiO_2_ (HR 1.744, 95% CI 1.355–2.245) was associated with an increased risk of 28-day mortality (Additional file 2: Table S6). Second, we changed the follow-up period to ensure all patients were ventilated during follow-up and found that hyperoxemia was associated with a 1.207-fold increase risk of death (HR 1.207, 95% CI 1.098–1.327), while high FiO_2_ was associated with 1.242-fold increase risk of death (HR 1.242, 95% CI 1.133–1.363) (Additional file 2: Table S7).

## Discussion

Time-varying intensity of oxygen exposure, as measured by daily TWA-PaO_2_ or TWA-FiO_2_, was associated with the increased 28-day mortality for patients with mechanical ventilation, and the strength of the association manifested predominantly in the early-middle course of illness. Importantly, we observed a cumulative effect of harmful exposure (TWA-PaO_2_ ≥ 120 mmHg or TWA-FiO_2_ ≥ 0.5) over time. The impact of high FiO_2_ on mortality was partly mediated by hyperoxemia, meanwhile, high FiO_2_ can also impact mortality directly.

Our findings that oxygen exposure is associated with mortality is generally in accordance with the results of previous studies [6, 18, 19], although some differences exist. First, the assessment period of oxygen exposure was different. Prior retrospective studies exploring the association between oxygen exposure and outcome are limited by using a single measure of PaO_2_ or FiO_2_, usually taken within 24 h after ICU admission. It is biologically implausible that a single measure of oxygen exposure could shift outcomes so dramatically. To address this limitation, we evaluated the oxygen exposure throughout the entire course of ICU admission (up to 28 days) and treated daily oxygen exposure as a time-varying exposure in PAMMs to explore the time-varying effects over time. Second, the threshold to define hyperoxemia and high FiO_2_ varied in previous studies, the threshold ranges from 100 to 200 mmHg for hyperoxemia and 0.5–1.0 for high FiO_2_ [20, 21], which simply from an empirical or biological standpoint, and remains unclear whether these values could provide the best measure to elucidate harm. From another aspect, we identified the PaO_2_ and FiO_2_ threshold above which the risk of 28-day mortality began to increase. Third, in previous observational studies, the multivariable regression model only adjusted for baseline confounders, which could lead to residual confounders, such as the postbaseline time-dependent patient differences. The difference in present study is that we employed PAMMs to account for both baseline and time-dependent confounders.

We visualized the longitudinal association between TWA-PaO_2_ or TWA-FiO_2_ and 28-day mortality throughout the entire course of ICU admission and suggested that clinicians should pay more attention to PaO_2_ and FiO_2_ during the early-middle course of illness (approximately 4–16 days after ICU admission). While for the high level of FiO_2_, the impact on mortality was persistent over time. Visual inspection showed that the optimum range for PaO_2_ was approximately 80–120 mmHg, and FiO_2_ should be kept as low as possible to sustain the target PaO_2_.

The current study has implications for interpreting recent randomized control trials (RCTs) evaluating the association between conservative oxygen targets and clinical outcomes. The Oxygen-ICU trial [22] found that oxygen supplementation titrated to more conservative oxygen targets (targeting a PaO_2_ of 70–100 mmHg during the ICU stay) was associated with improved outcomes compared with conventional oxygen targets (allowing a PaO_2_ up to 150 mmHg during the ICU stay), with no mention about the proportion of patients exposed to hyperoxemia. The LOCO_2_ trial [23] assigned patients with acute respiratory distress syndrome to receive either conservative oxygen therapy (target PaO_2_, 50–70 mmHg) or liberal oxygen therapy (target PaO_2_, 90–105 mmHg) for 7 days and found that conservative oxygen therapy did not increase survival at 28 days, while was associated with mesenteric ischemic events. ﻿Recently, a Dutch RCT conducted in ICU patients fulfilling the systemic inflammatory response syndrome criteria found no significant difference between high-normal (14–18 kPa) and low-normal (targeting a PaO_2_ of 8–12 kPa) oxygenation targets for non-respiratory organ dysfunction over the first 14 days [24]. The HOT-ICU trial [25] randomly assigned patients with acute hypoxemic respiratory failure to receive oxygen therapy targeting a PaO_2_ of either 60 mmHg (lower-oxygenation group) or 90 mmHg (higher-oxygenation group) for a maximum of 90 days and declared that a lower-oxygenation target did not result in lower mortality than a higher target at 90 days. These RCTs are unlikely to represent the frequency and persistence of hyperoxemia in clinical practice. In our research, the median TWA-PaO_2_ was 130.5 mmHg, and it seems that a more conservative strategy in the early phase of disease would demonstrate a clinical benefit. However, a PaO_2_ target that is too conservative may expose patients to harmful hypoxemia, as was likely the case in the LOCO_2_ trial.

Oxygen exposure-induced oxidative stress was time- and dose-dependent [26, 27], and the assessment of cumulative exposure was equally important. We concluded that a time- and dose-dependent exposure to hyperoxemia were both associated with harm, which was consistent with two studies [19, 28] and at odds with one prior study. ﻿Palmer et al. [29] defined hyperoxemia dose as the area between the PaO_2_ time curve and a boundary of 100 mmHg divided by the hours of potential exposure (24, 72, 120, or 168 h) and did not observe a dose–response relationship. The inconsistencies are probably attributable, at least partly, to the different threshold values of hyperoxemia and exposure window used.

Limited data was available regarding the cumulative effect of exposure to high FiO_2_ on mortality, which was also restricted by a single measurement or a shorter exposure window [30–32]. According to 73,992 patients undergoing non-cardiothoracic surgery, Staehr-Rye et al. [33] found that high intraoperative FiO_2_ (0.79, range 0.64–1.0) was associated in a dose-dependent manner with major respiratory complications and 30-day mortality. In the present study, the cumulative effect of exposure to high FiO_2_ (TWA-FiO_2_ ≥ 0.5) on mortality during the entire course of ICU admission was significant. Besides, we also found that the higher FiO_2_ could directly impact the mortality, independently from PaO_2_, which supports the causal relationship between higher FiO_2_ and mortality.

Several limitations to the present study should be considered. First, the design of our study is a retrospective observational study, we considered only segmental measured confounders, and the residual measured confounders and unmeasured confounders cannot be fully included. Second, we included all patients who received mechanical ventilation, which makes the results more generalizable. Consequently, significant heterogeneity might exist between groups [34]. We performed several subgroup analyses based on initial admission diagnosis to account for this limitation. Further research is required to explore the impact of time-varying oxygen exposure on mortality in specific patients. Finally, the MIMIV-IV, like all databases with routinely collected data, comprises records with missing values. However, the results from our primary analysis were robust in sensitivity analyses after excluding the missing data.

## Conclusion

In conclusion, PaO_2_ and FiO_2_ should be carefully monitored in patients with mechanical ventilation, especially during the early-middle course after ICU admission. Cumulative exposure to higher intensities of oxygen exposure was associated with an increased risk of death. Additionally, high FiO_2_ can impact mortality directly, independently from hyperoxemia.

## Supplementary Information

**Additional file 1. **: Codes for piece-wise exponential additive mixed models

**Additional file 2. Table S1**: Baseline characteristics and outcomes of patients stratified by TWA-PaO2 on Day 1; **Table S2**: Baseline characteristics and outcomes of patients stratified by TWA-FiO_2_ on Day 1; **Table S3**: Effect of time-varying TWA-PaO_2_ and TWA-FiO_2_ on 28-day mortality of 7784 patients with mechanical ventilation; **Table S4**: Effect of time spent with hyperoxemia and high FiO_2_ on 28-day mortality of 7784 patients with mechanical ventilation; **Table S5**: Effect of cumulative exposure of hyperoxemia and high FiO_2_ on 28-day mortality of 7784 patients with mechanical ventilation; **Table S6**: Effect of time-varying hyperoxemia and high FiO_2_ on 28-day mortality after excluding missing data of daily TWA-PaO_2_ or TWA-FiO_2_; **Table S7**: Effect of time-varying hyperoxemia and high FiO_2_ on 28-day mortality in patients with mechanical ventilation, patients were followed up from inclusion until death, liberated from mechanical ventilation, ICU discharge, or 28 days in the ICU, whichever occurred first; **Figure S1**: Study flow diagram in present study.** Figure S2**: Time-varying effect of TWA-PaO_2_ (A) or TWA-FiO_2_ (B) on 28-day mortality

## Data Availability

The datasets presented in the current study are available in the MIMIC-IV database (https://physionet.org/content/mimiciv/2.0/).

## Abbreviations

ICU: Intensive care units
PaO_2_: Arterial oxygen tension
FiO_2_: Fraction of inspired oxygen
MIMIC-IV: Medical information mart for intensive care IV
ECMO: Extracorporeal membrane oxygenation
SOFA: Sequential organ failure assessment
SAPS II: Simplified acute physiology score II
OASIS: Oxford acute severity of illness score
TWA: Time-weighted average
VFDs: Ventilation-free days
PAMMs: Piece-wise exponential additive mixed models
MICE: Multiple imputations by chained equation
CMA: Causal mediation analysis
RCTs: Randomized control trials
