# Supplementary material for: Time-varying intensity of oxygen exposure is associated with mortality in critically ill patients with mechanical ventilation
Source: Crit Care. 2022 Aug 5;26:239. doi: 10.1186/s13054-022-04114-w (PMC9356484; doi:10.1186/s13054-022-04114-w)
Supplement: Supplementary file 1 — Additional file 1. : Codes for piece-wise exponential additive mixed models [file 13054_2022_4114_MOESM1_ESM.docx]

**Codes for** **piece-wise exponential additive mixed models (Figure 1)**

gam<- gam(formula =endpt~admission_age +

gender+admissiontype + charlson_comorbidity_index +

weight_admit + mv_status+dyna_sofa+ max_pco2 +

vaso_use+te(tend,twa_pao2),

data = total_data,

family = binomial(),offset = offset)

time_df<-total_data%>%

make_newdata(twa_pao2 = unique(twa_pao2)) %>%

add_term(gam, term = "twa_pao2")

time_gg<-ggplot(time_df, aes(x = twa_pao2)) +

geom_line(aes(y = exp(fit)),colour="#990000",lwd = 1.1) +

geom_ribbon(aes(ymin = exp(ci_lower), ymax = exp(ci_upper)),

alpha = .2,fill = "#FC8D59") +

ylab("Hazard ratio for 28-day mortality")+xlab("Time varying TWA-PaO2 (mmHg)")+

coord_cartesian(ylim = c(0,20)) +

scale_x_continuous(breaks = c(0,100,200,300,400))+

theme(panel.background = element_rect(fill = "white"),

legend.key= element_rect(fill= "white"),

axis.line.x = element_line(colour = "black", size = 1),

axis.line.y = element_line(colour = "black", size = 1))+ theme(legend.position="none")+

theme(axis.title=element_text(size = rel(1.2)))+

theme(axis.text=element_text(size = rel(1.2)))+

geom_hline(yintercept = 1,linetype='dashed',colour='black',size=0.4)

time_df_1<-total_data%>%

make_newdata(tend=c(7,14,21),twa_pao2 = seq(40, 400, by = 10)) %>%

add_term(gam, term = "twa_pao2")

time_gg_1<-ggplot(time_df_1, aes(x = twa_pao2)) +

geom_line(aes(y = exp(fit), col = factor(tend)), lwd = 1.1) +

geom_ribbon(aes(ymin = exp(ci_lower), ymax = exp(ci_upper), fill = factor(tend)),

alpha = .2) +

scale_color_manual(name = "Days", values = c("#FDBB84","#EF6548","#7F0000")) +

scale_fill_manual(name = "Days", values = c("#FDBB84","#EF6548","#7F0000"))+ coord_cartesian(ylim = c(0, 25)) +

theme(panel.background = element_rect(fill = "white"),

legend.key= element_rect(fill= "white"),

axis.line.x = element_line(colour = "black", size = 1),

axis.line.y = element_line(colour = "black", size = 1))+

geom_hline(yintercept = 1,linetype='dashed',colour='black',size=0.4)+

theme(legend.position="top")+theme(axis.title=element_text(size = rel(1.2)))+

theme(axis.text=element_text(size = rel(1.2)))+ylab("Hazard ratio for 28-day mortality")+xlab("Time varying TWA-PaO2 (mmHg)")

te_df<- total_data%>%

make_newdata(tend = unique(tend)) %>%

add_term(gam, term = "twa_pao2")

df_gg<-ggplot(te_df, aes(x = tend, y = exp(fit))) +

geom_step(lwd = 1.1,color="#990000") +

geom_stepribbon(aes(ymin = exp(ci_lower), ymax = exp(ci_upper)),

alpha = .2,fill="#FC8D59") +

ylab("Hazard ratio for 28-day mortality") +

xlab("Days after ICU admission (days)")+theme_classic()+

scale_x_continuous(breaks = c(0,4,8,12,16,20,24,28))+

theme(panel.background = element_rect(fill = "white"),

legend.key= element_rect(fill= "white"),

axis.line.x = element_line(colour = "black", size = 1),

axis.line.y = element_line(colour = "black", size = 1))+

geom_hline(yintercept = 1,linetype='dashed',colour='black',size=0.4)+

theme(axis.title=element_text(size = rel(1.2)))+

theme(axis.text=element_text(size = rel(1.2)))

te_df_1<-total_data%>%

make_newdata(tend = unique(tend),twa_pao2 = c(100,200,300)) %>%

add_term(gam, term = "twa_pao2")

df_gg_1<-ggplot(te_df_1, aes(x = tend, y = exp(fit))) +

geom_step(aes(col = factor(twa_pao2)), lwd = 1.1) +

geom_stepribbon(aes(ymin = exp(ci_lower), ymax = exp(ci_upper),fill=factor(twa_pao2)),

alpha = .2) +

scale_color_manual(

name = expression(TWA-PaO2),

values = c("#FDBB84","#EF6548","#7F0000")) +

scale_fill_manual(

name = expression(TWA-PaO2),

values = c("#FDBB84","#EF6548","#7F0000")) +

ylab("Hazard ratio for 28-day mortality") +

xlab("Days after ICU admission (days)")+theme(legend.position = "bottom")+theme_classic()+

scale_x_continuous(breaks = c(0,4,8,12,16,20,24,28))+

theme(panel.background = element_rect(fill = "white"),

legend.key= element_rect(fill= "white"),

axis.line.x = element_line(colour = "black", size = 1),

axis.line.y = element_line(colour = "black", size = 1))+

theme(legend.position="top")+theme(axis.title=element_text(size = rel(1.2)))+

theme(axis.text=element_text(size = rel(1.2)))+geom_hline(yintercept = 1,linetype='dashed',colour='black',size=0.4)

p_total<-plot_grid(time_gg, time_gg_1,df_gg,df_gg_1,ncol=2,labels=LETTERS[1:4],align=c("v","h"))
