# Supplementary material for: Time-varying intensity of oxygen exposure is associated with mortality in critically ill patients with mechanical ventilation
Source: Crit Care. 2022 Aug 5;26:239. doi: 10.1186/s13054-022-04114-w (PMC9356484; doi:10.1186/s13054-022-04114-w)

Table S1: ﻿﻿Baseline characteristics and outcomes of patients stratified by TWA-PaO_2_ on Day 1

|  | ﻿Stratification by TWA-PaO_2_ on Day 1^*^ | | | P value |
| --- | --- | --- | --- | --- |
|  | ≤80 mmHg  (n=528) | 80-120 mmHg  (n=2213) | ﻿≥120 mmHg  (n=3803) |  |
| Age (years) | 66.0 (55.0-75.0) | 65.0 (54.0-75.0) | 65.0 (53.0-76.0) | 0.69 |
| Male (gender) | 294 (55.7) | 1294 (58.5) | 2202 (57.9) | 0.51 |
| Weight (kg) | 86.2 (68.9-106.8) | 85.5 (70.0-102.0) | 78.2 (65.9-94.0) | <0.001 |
| Height (cm) | 168.0 (160.0-178.0) | 170.0 (163.0-178.0) | 170.0 (163.0-178.0) | 0.26 |
| **Admission type, n (%)** | | | | <0.001 |
| Medical | 281 (53.2) | 1240 (56.0) | 2414 (63.5) |  |
| Surgical elective | 3 (0.6) | 30 (1.4) | 115 (3.0) |  |
| Surgical urgent | 165 (31.2) | 642 (29.0) | 742 (19.5) |  |
| Other | 79 (15.0) | 301 (13.6) | 532 (14.0) |  |
| **Ethnicity, n (%)** | | | | 0.043 |
| White | 310 (58.7) | 1352 (61.1) | 2297 (60.4) |  |
| Black | 68 (12.9) | 201 (9.1) | 366 (9.6) |  |
| Hispanic | 17 (3.2) | 70 (3.2) | 141 (3.7) |  |
| Other | 120 (22.7) | 546 (24.7) | 882 (23.2) |  |
| Elixhauser comorbidity score | 6.0 (4.0-8.0) | 6.0 (4.0-8.0) | 6.0 (4.0-8.0) | 0.20 |
| **Severity of illness** | | | | |
| GCS | 14.5 (1.4) | 14.5 (1.2) | 14.6 (1.1) | 0.29 |
| SAPS II | 46.0 (36.8-56.0) | 46.0 (37.0-57.0) | 43.0 (34.0-54.0) | <0.001 |
| OASIS | 44.0 (38.0-50.0) | 43.0 (37.0-48.0) | 41.0 (36.0-47.0) | <0.001 |
| SOFA | 4.0 (3.0-5.0) | 4.0 (3.0-5.0) | 3.0 (2.0-5.0) | 0.001 |
| Use of vasopressor (1 st 24h) | 289 (54.7) | 1409 (63.7) | 2371 (62.3) | 0.001 |
| **Initial diagnosis, n (%)** | | | | |
| Sepsis | 504 (95.5) | 2113 (95.5) | 3419 (89.9) | <0.001 |
| Acute respiratory failure | 527 (99.8) | 2122 (95.9) | 1289 (33.9) | <0.001 |
| Cardiac arrest | 27 (5.1) | 135 (6.1) | 292 (7.7) | 0.015 |
| **Vital signs (**1 st 24h**)** | | | | |
| Heart rate (bpm) | 88.7 (76.4-101.5) | 87.7 (75.6-100.6) | 86.3 (75.3-99.1) | 0.016 |
| MAP (mmHg) | 74.1 (69.4-81.1) | 74.7 (69.8-81.1) | 76.5 (70.8-83.4) | <0.001 |
| Temperature (℃) | 37.1 (36.8-37.6) | 37.1 (36.7-37.5) | 37.0 (36.6-37.4) | <0.001 |
| **Parameters of mechanical ventilation (**1 st 24h**)** | | | | |
| TWA-FiO_2_ | 0.57 (0.48-0.72) | 0.53 (0.45-0.65) | 0.49 (0.42-0.57) | <0.001 |
| Total respiratory rate (bpm) | 21.9 (18.7-25.0) | 21.2 (18.5-24.2) | 19.7 (17.2-22.3) | <0.001 |
| Tidal volume (ml/kg) | 6.8 (6.0-7.7) | 7.0 (6.4-7.7) | 7.2 (6.5-8.0) | <0.001 |
| PEEP (cmH_2_O) | 8.0 (6.3-10.6) | 7.2 (5.5-9.3) | 5.7 (5.1-7.5) | <0.001 |
| Plateau pressure (cmH_2_O) | 21.4 (18.7-24.9) | 20.1 (17.6-23.1) | 18.2 (15.8-21.2) | <0.001 |
| Use of NMBA, n (%) | 55 (10.4) | 152 (6.9) | 200 (5.3) | <0.001 |
| **Laboratory data (**1 st 24h**)** | | | | |
| pH | 7.4 (7.3-7.4) | 7.3 (7.3-7.4) | 7.4 (7.3-7.4) | <0.001 |
| PaCO_2_ (mmHg) | 44.4 (38.5-54.5) | 41.0 (35.8-47.0) | 38.8 (34.5-43.0) | <0.001 |
| TWA-PaO_2_ (mmHg) | 72.0 (66.0-76.5) | 100.5 (91.6-110.0) | 163.0 (139.7-197.5) | <0.001 |
| Lactate (mmol/L) | 1.6 (1.1-2.5) | 1.9 (1.3-3.1) | 2.1 (1.4-3.5) | <0.001 |
| Bicarbonate (mmol/L) | 24.5 (20.5-29.0) | 22.0 (18.8-25.3) | 22.0 (19.3-24.5) | <0.001 |
| Fluid balance in the first 24 h (L) | 1.04 (-0.51-3.74) | 1.79 (-0.14-4.77) | 2.0 (0-5.49) | <0.001 |
| **Clinical outcomes** | | | | |
| 28-day mortality, n (%) | 170 (32.2) | 628 (28.4) | 970 (25.5) | 0.001 |
| In-hospital mortality, n (%) | 173 (32.8) | 647 (29.2) | 1044 (27.5) | 0.025 |
| Alive and VFDs (days) | 15.6 (0.0-23.0) | 18.6 (0.0-23.8) | 20.2 (0.0-24.3) | <0.001 |
| Length of ICU stay (days) | 8.6 (5.1-14.0) | 8.5 (5.1-13.6) | 8.0 (5.1-13.2) | 0.158 |
| Length of hospital stay (days) | 14.0 (8.0-22.0) | 14.0 (9.0-22.0) | 15.0 (9.0-24.0) | 0.005 |

GCS: Glasgow Coma Scale; SAPS II: Simplified acute physiology score II; OASIS: Oxford Acute Severity of Illness Score; SOFA: sequential organ failure assessment; MAP: mean arterial pressure; TWA: Time-weighted average; FiO_2_: Fraction of inspired oxygen; PEEP: Positive end-expiratory pressure; NMBA: neuromuscular blocking agent;

PCO_2_: partial pressure of carbon dioxide; PO_2_: partial pressure of oxygen; VFDs: Ventilation-free days; ICU: Intensive Care Unit.

Table S2: ﻿﻿Baseline characteristics and outcomes of patients stratified by TWA-FiO_2_ on Day 1

|  | Stratification by TWA-FiO_2_ on Day 1^*^ | | | P value |
| --- | --- | --- | --- | --- |
|  | ≤ 0.30  (n=132) | 0.30-0.50  (n=3543) | ﻿≥ 0.50  (n=4103) |  |
| Age (years) | 63.5 (47.8-78.0) | 67.0 (54.0-77.0) | 64.0 (53.0-75.0) | <0.001 |
| Male (gender) | 62 (47.0) | 1969 (55.6) | 2417 (58.9) | 0.001 |
| Weight (kg) | 73.1 (63.6-85.1) | 77.0 (64.0-92.0) | 83.9 (69.3-100.7) | <0.001 |
| Height (cm) | 168.0 (157.0-174.0) | 168.0 (160.0-178.0) | 170.0 (163.0-178.0) | <0.001 |
| **Admission type, n (%)** | | | | <0.001 |
| Medical | 65 (49.2) | 2230 (62.9) | 2416 (58.9) |  |
| Surgical elective | 0 (0.0) | 38 (1.1) | 115 (2.8) |  |
| Surgical urgent | 46 (34.8) | 763 (21.5) | 1019 (24.8) |  |
| Other | 21 (15.9) | 512 (14.5) | 553 **(13.5)** |  |
| **Ethnicity, n (%)** | | | | <0.001 |
| White | 60 (45.5) | 2040 (57.6) | 2590 (63.1) |  |
| Black | 18 (13.6) | 427 (12.1) | 378 (9.2) |  |
| Hispanic | 8 (6.1) | 108 (3.0) | 150 (3.7) |  |
| Other | 41 (31.1) | 854 (24.1) | 891 (21.7) |  |
| Elixhauser comorbidity score | 5.0 (3.0-8.0) | 6.0 (4.0-8.0) | 6.0 (4.0-8.0) | 0.080 |
| **Severity of illness** | | | | |
| GCS | 14.6 (1.0) | 14.6 (1.1) | 14.5 (1.2) | 0.196 |
| SAPS II | 35.5 (27.0-43.2) | 40.0 (31.0-51.0) | 45.0 (36.0-56.0) | <0.001 |
| OASIS | 40.0 (34.0-45.0) | 41.0 (35.0-46.0) | 43.0 (37.0-49.0) | <0.001 |
| SOFA | 3.0 (2.0-4.0) | 3.0 (2.0-5.0) | 4.0 (3.0-6.0) | <0.001 |
| Use of vasopressor (1 st 24h) | 40 (30.3) | 1703 (48.1) | 2717 (66.2) | <0.001 |
| **Initial diagnosis, n (%)** |  |  |  |  |
| Sepsis | 105 (79.5) | 3066 (86.5) | 3787 (92.3) | <0.001 |
| Acute respiratory failure | 10 (7.6) | 1041 (29.4) | 2887 (70.4) | <0.001 |
| Cardiac arrest | 2 (1.5) | 158 (4.5) | 333 (8.1) | <0.001 |
| **Vital signs (**1 st 24h**)** | | | | |
| Heart rate (bpm) | 83.6 (73.4-94.1) | 84.7 (73.9-97.1) | 88.1 (76.5-101.3) | <0.001 |
| MAP (mmHg) | 76.4 (70.4-85.9) | 76.8 (71.0-83.8) | 75.1 (69.8-81.5) | <0.001 |
| Temperature (℃) | 37.0 (36.7-37.3) | 37.0 (36.7-37.4) | 37.0 (36.7-37.5) | 0.694 |
| **Parameters of mechanical ventilation (**1 st 24h**)** | | | | |
| TWA-FiO_2_ | 0.30 (0.30-0.30) | 0.42 (0.40-0.45) | 0.58 (0.52-0.68) | <0.001 |
| Total respiratory rate (bpm) | 18.7 (15.9-21.3) | 19.5 (17.0-22.1) | 20.8 (18.2-23.8) | <0.001 |
| Tidal volume (ml/kg) | 6.7 (6.1-7.4) | 6.9 (6.2-7.7) | 7.1 (6.5-7.9) | <0.001 |
| PEEP (cmH_2_O) | 5.1 (5.0-5.8) | 5.3 (5.0-6.6) | 7.2 (5.4-9.3) | <0.001 |
| Plateau pressure (cmH_2_O) | 16.9 (14.1-20.1) | 17.4 (15.0-19.9) | 20.3 (17.5-23.3) | <0.001 |
| Use of NMBA, n (%) | 1 (0.8) | 93 (2.6) | 319 (7.8) | <0.001 |
| **Laboratory data (**1 st 24h**)** | | | | |
| pH | 7.4 (7.4-7.4) | 7.4 (7.3-7.4) | 7.3 (7.3-7.4) | <0.001 |
| PaCO_2_ (mmHg) | 36.5 (34.0-41.9) | 38.0 (34.0-43.3) | 41.0 (36.3-46.1) | <0.001 |
| TWA-PaO_2_ (mmHg) | 117.7 (97.0-149.8) | 142.0 (110.0-179.5) | 121.7 (95.2-163.5) | <0.001 |
| Lactate (mmol/L) | 1.4 (1.1-1.9) | 1.8 (1.2-2.9) | 2.2 (1.4-3.6) | <0.001 |
| Bicarbonate (mmol/L) | 23.1 (20.6-26.0) | 22.3 (19.6-25.5) | 22.0 (19.2-25.0) | 0.001 |
| Fluid balance in the first 24 h (L) | 0.3 (-0.69-1.72) | 1.09 (-0.46-3.74) | 2.03 (0-5.4) | <0.001 |
| **Clinical outcomes** | | | | |
| 28-day mortality, n (%) | 29 (22.0) | 906 (25.6) | 1108 (27.0) | 0.192 |
| In-hospital mortality, n (%) | 27 (20.5) | 949 (26.8) | 1177 (28.7) | 0.031 |
| Alive and VFDs (days) | 20.8 (9.2-24.6) | 20.8 (0.0-24.5) | 19.0 (0.0-24.0) | <0.001 |
| Length of ICU stay (days) | 6.0 (4.2-11.7) | 7.2 (4.7-12.3) | 8.3 (5.2-13.8) | <0.001 |
| Length of hospital stay (days) | 9.0 (5.8-18.0) | 13.0 (8.0-22.0) | 14.0 (9.0-23.0) | <0.001 |

GCS: Glasgow Coma Scale; SAPS II: Simplified acute physiology score II; OASIS: Oxford Acute Severity of Illness Score; SOFA: sequential organ failure assessment; MAP: mean arterial pressure; TWA: Time-weighted average; FiO_2_: Fraction of inspired oxygen; PEEP: Positive end-expiratory pressure; NMBA: neuromuscular blocking agent;

PCO_2_: partial pressure of carbon dioxide; PO_2_: partial pressure of oxygen; VFDs: Ventilation-free days; ICU: Intensive Care Unit.

Table S3: Effect of time-varying TWA-PaO_2_ and TWA-FiO_2_ on 28-day mortality of 7784 patients with mechanical ventilation.

|  | TWA-PaO_2_ | | TWA-FiO_2_ | |
| --- | --- | --- | --- | --- |
|  | HR (95% CI) | P value | HR (95% CI) | P value |
| **Baseline variables** | | | | |
| Age, years | 1.019 (1.015-1.022) | <0.001 | 1.019 (1.016-1.023) | <0.001 |
| Male | 0.974 (0.886-1.071) | 0.59 | 0.976 (0.888-1.074) | 0.62 |
| Admission type | | | | |
| Medical | Reference | -- | Reference | -- |
| Surgical elective | 0.236 (0.149-0.374) | <0.001 | 0.246 (0.155-0.390) | <0.001 |
| Surgical urgent | 0.861 (0.772-0.959) | 0.0066 | 0.812 (0.729-0.906) | <0.001 |
| Other | 0.655 (0.568-0.755) | <0.001 | 0.649 (0.562-0.748) | <0.001 |
| Weight, kg | 0.993 (0.991-0.995) | <0.001 | 0.992 (0.990-0.994) | <0.001 |
| Elixhauser comorbidity score | 1.033 (1.014-1.052) | <0.001 | 1.037 (1.018-1.057) | <0.001 |
| **Time-varying variables** | | | | |
| Receiving IMV | 0.406 (0.365-0.452) | <0.001 | 0.446 (0.401-0.497) | <0.001 |
| Use of vasopressor | 1.321 (1.179-1.481) | <0.001 | 1.318 (1.175-1.477) | <0.001 |
| SOFA score | 1.151 (1.136-1.167) | <0.001 | 1.133 (1.112-1.148) | <0.001 |
| PaCO_2_, mmHg | 1.019 (1.0157-1.023) | <0.001 | 1.013 (1.001-1.017) | <0.001 |
| TWA-PaO_2_ per 5mmHg | 1.801 (1.585-2.046) | <0.001 | -- | -- |
| TWA-FiO_2_ per 0.05 | -- | -- | 2.688 (2.407-3.002) | <0.001 |

HR: Hazard ratio; CI: Confidence interval;IMV: Invasive mechanical ventilation; SOFA: sequential organ failure assessment; TWA: Time-weighted average; PCO_2_: partial pressure of carbon dioxide; FiO_2_: Fraction of inspired oxygen.

Table S4: ﻿Effect of time spent with hyperoxemia and high FiO_2_ on 28-day mortality of 7784 patients with mechanical ventilation.

|  | Exposure to hyperoxemia | | Exposure to high FiO_2_ | |
| --- | --- | --- | --- | --- |
|  | HR (95% CI) | P value | HR (95% CI) | P value |
| Proportion of time spent in  hyperoxemia, per 5% | 1.412 (1.302-1.531) | <0.001 | -- | -- |
| Proportion of time spent in  high FiO_2_, per 5% | -- | -- | 1.284 (1.204-1.369 | <0.001 |
| **Baseline variables** | | | | |
| Age, years | 1.019 (1.015-1.023) | <0.001 | 1.018 (1.014-1.022) | <0.001 |
| Male | 0.976 (0.887-1.072) | 0.61 | 0.972 (0.884-1.068) | 0.55 |
| Admission type | | | | |
| Medical | Reference |  | Reference |  |
| Surgical elective | 0.243 (0.155-0.381) | <0.001 | 0.243 (0.155-0.380) | <0.001 |
| Surgical urgent | 0.873 (0.784-0.974) | 0.015 | 0.829 (0.744-0.924) | <0.001 |
| Other | 0.669 (0.580-0.771) | <0.001 | 0.644 (0.559-0.743) | <0.001 |
| Weight, kg | 0.994 (0.992-0.996) | <0.001 | 0.992 (0.989-0.994) | <0.001 |
| Elixhauser comorbidity score | 1.034 (1.015-1.053) | <0.001 | 1.039 (1.020-1.058) | <0.001 |
| **Time-varying variables** | | | | |
| Receiving IMV | 0.421 (0.378-0.468) | <0.001 | 0.424 (0.381-0.472) | <0.001 |
| Use of vasopressor | 1.358 (1.212-1.521) | <0.001 | 1.383 (1.235-1.550) | <0.001 |
| SOFA score | 1.153 (1.138-1.169) | <0.001 | 1.131 (1.116-1.146) | <0.001 |
| PaCO_2_, mmHg | 1.017 (1.014-1.021) | <0.001 | 1.014 (1.011-1.018) | <0.001 |

HR: Hazard ratio; CI: Confidence interval; IMV: Invasive mechanical ventilation; SOFA: sequential organ failure assessment; TWA: Time-weighted average; PCO_2_: partial pressure of carbon dioxide; FiO_2_: Fraction of inspired oxygen.

Table S5: ﻿Effect of cumulative exposure of hyperoxemia and high FiO_2_ on 28-day mortality of 7784 patients with mechanical ventilation.

|  | Exposure to hyperoxemia | | Exposure to high FiO_2_ | |
| --- | --- | --- | --- | --- |
|  | HR (95% CI) | P value | HR (95% CI) | P value |
| Cumulative exposure to hyperoxemia | 1.0014 (1.001-1.0017) | <0.001 | -- | -- |
| Cumulative exposure to high FiO_2_ | -- | -- | 1.003 (1.0023-1.0036) | <0.001 |
| **Baseline variables** | | | | |
| Age, years | 1.019 (1.015-1.023) | <0.001 | 1.018 (1.015-1.022) | <0.001 |
| Male | 0.980 (0.892-1.078) | 0.68 | 0.990 (0.9902-1.088) | 0.83 |
| Admission type | | | | |
| Medical | Reference |  | Reference |  |
| Surgical elective | 0.244 (0.156-0.382) | <0.001 | 0.251 (0.160-0.393) | <0.001 |
| Surgical urgent | 0.867 (0.778-0.966) | 0.0097 | 0.826 (-/742-0.920) | <0.001 |
| Other | 0.664 (0.576-0.766) | <0.001 | 0.638 (0.553-0.735) | <0.001 |
| Weight, kg | 0.993 (0.991-0.995) | <0.001 | 0.992 (0.990-0.994) | <0.001 |
| Elixhauser comorbidity score | 1.034 (1.014-1.053) | <0.001 | 1.037 (1.018-1.057) | <0.001 |
| **Time-varying variables** | | | | |
| Receiving IMV | 0.413 (0.372-0.460) | <0.001 | 0.414 (0.372-0.460) | <0.001 |
| Use of vasopressor | 1.349 (1.204-1.511) | <0.001 | 1.387 (1.238-1.554) | <0.001 |
| SOFA score | 1.149 (1.133-1.164) | <0.001 | 1.134 (1.112-1.150) | <0.001 |
| PaCO_2_, mmHg | 1.017 (1.013-1.020) | <0.001 | 1.014 (1.010-1.017) | <0.001 |

HR: Hazard ratio; CI: Confidence interval; IMV: Invasive mechanical ventilation; SOFA: sequential organ failure assessment; TWA: Time-weighted average; PCO_2_: partial pressure of carbon dioxide; FiO_2_: Fraction of inspired oxygen.

Table S6: Effect of time-varying hyperoxemia and high FiO_2_ on 28-day mortality after excluding missing data of daily TWA-PaO_2_ or TWA-FiO_2._

|  | Exposure to hyperoxemia | | Exposure to high FiO_2_ | |
| --- | --- | --- | --- | --- |
|  | HR (95% CI) | P value | HR (95% CI) | P value |
| **Baseline variables** | | | | |
| Age, years | 1.013 (1.007-1.018) | <0.001 | 1.011 (1.0009-1.020) | 0.031 |
| Male | 0.968 (0.834-1.123) | 0.66 | 0.910 (0.713-1.161) | 0.45 |
| Admission type | | | | |
| Medical | Reference |  | Reference |  |
| Surgical elective | 0.278 (0.150-0.511) | <0.001 | 0.196 (0.048-0.811) | 0.024 |
| Surgical urgent | 0.948 (0.806-1.114) | 0.52 | 0.967 (0.747-1.253) | 0.80 |
| Other | 0.664 (0.531-0.830) | <0.001 | 0.883 (0.592-1.316) | 0.54 |
| Weight, kg | 0.994 (0.991-0.997) | <0.001 | 0.994 (0.990-0.999) | 0.017 |
| Elixhauser comorbidity score | 1.0 (0.971-1.031) | 0.98 | 0.996 (0.946-1.047) | 0.86 |
| **Time-varying variables** | | | | |
| Receiving IMV | 0.305 (0.250-0.373) | <0.001 | 0.173 (0.119-0.251) | <0.001 |
| Use of vasopressor | 2.240 (1.870-2.683) | <0.001 | 2.762 (1.996-3.821) | <0.001 |
| SOFA score | 1.184 (1.161-1.207) | <0.001 | 1.176 (1.138-1.216) | <0.001 |
| PaCO_2_, mmHg | 1.024 (1.019-1.029) | <0.001 | 1.018 (1.009-1.026) | <0.001 |
| Any hyperoxemia  (TWA-PaO_2_≥120 mmHg) | 1.232 (1.059-1.435) | 0.0070 | -- | -- |
| Any high FiO_2_  (TWA-FiO_2_ ≥ 0.5) | -- | -- | 1.744 (1.355–2.245) | <0.001 |

HR: Hazard ratio; CI: Confidence interval; IMV: Invasive mechanical ventilation; SOFA: sequential organ failure assessment; TWA: Time-weighted average; PCO_2_: partial pressure of carbon dioxide; FiO_2_: Fraction of inspired oxygen.

Table S7: Effect of time-varying hyperoxemia and high FiO2 on 28-day mortality in patients with mechanical ventilation, patients were followed up from inclusion until ﻿death, liberated from mechanical ventilation, ICU discharge, or 28 days in the ICU, whichever occurred first.

|  | Exposure to hyperoxemia | | Exposure to high FiO_2_ | |
| --- | --- | --- | --- | --- |
|  | HR (95% CI) | P value | HR (95% CI) | P value |
| **Baseline variables** | | | | |
| Age, years | 1.017 (1.014-1.021) | <0.001 | 1.017 (1.014-1.021) | <0.001 |
| Male | 0.967 (0.880-1.062) | 0.48 | 0.964 (0.877-1.059) | 0.44 |
| Admission type | | | | |
| Medical | Reference |  | Reference |  |
| Surgical elective | 0.341 (0.217-0.536) | 0.0030 | 0.345 (0.220-0.542) | <0.001 |
| Surgical urgent | 0.875 (0.785-0.975) | 0.016 | 0.859 (0.771-0.958) | 0.0061 |
| Other | 0.725 (0.629-0.836) | <0.001 | 0.720 (0.624-0.830) | <0.001 |
| Weight, kg | 0.994 (0.992-0.996) | <0.001 | 0.993 (0.991-0.995) | <0.001 |
| Elixhauser comorbidity score | 1.047 (1.028-1.066) | <0.001 | 1.049 (1.030-1.068) | <0.001 |
| **Time-varying variables** | | | | |
| Use of vasopressor | 1.386 (1.243-1.545) | <0.001 | 1.393 (1.250-1.553) | <0.001 |
| SOFA score | 1.091 (1.076-1.106) | <0.001 | 1.083 (1.069-1.098) | <0.001 |
| PaCO_2_, mmHg | 1.006 (1.003-1.011) | <0.001 | 1.0045 (1.0006-1.0008) | 0.024 |
| Any hyperoxemia  (TWA-PaO_2_≥120 mmHg) | 1.207 (1.098-1.327) | <0.001 | -- | -- |
| Any high FiO_2_  (TWA-FiO_2_ ≥ 0.5) | -- | -- | 1.242 (1.133-1.363) | <0.001 |

HR: Hazard ratio; CI: Confidence interval; SOFA: sequential organ failure assessment; TWA: Time-weighted average; PCO_2_: partial pressure of carbon dioxide; FiO_2_: Fraction of inspired oxygen.

Figure S1: Study flow diagram in present study.

Figure S2: Time-varying effect of TWA-PaO_2_ (A) or TWA-FiO_2_ (B) on 28-day mortality.


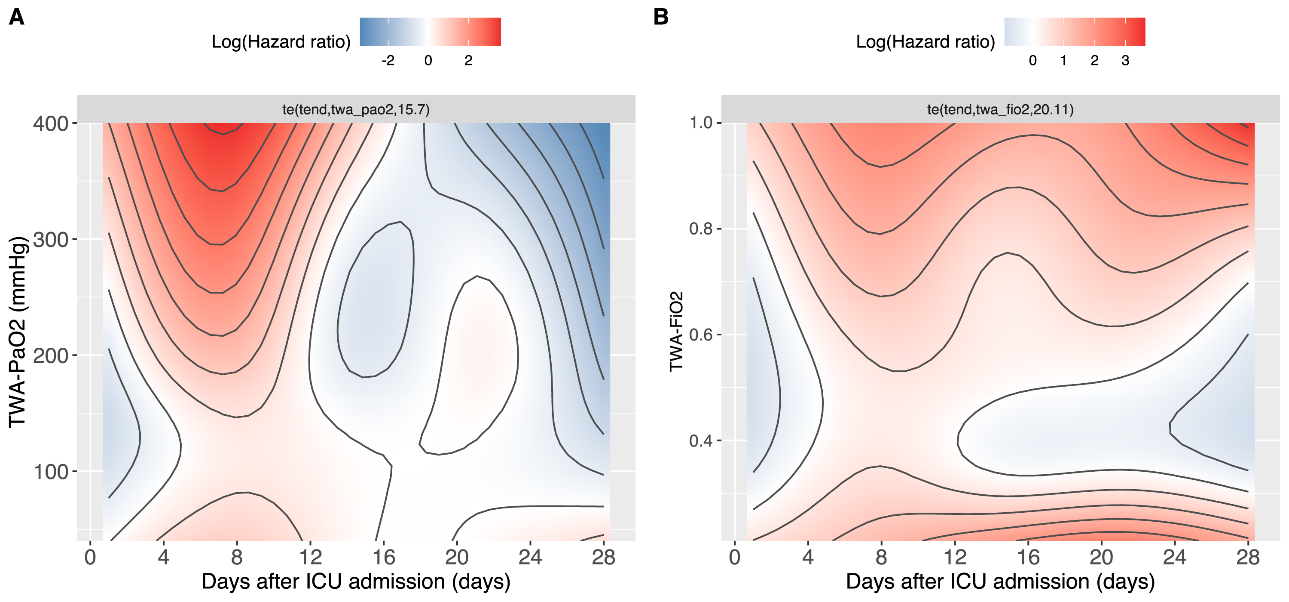

Supplement: Supplementary file 2 — Additional file 2. Table S1: Baseline characteristics and outcomes of patients stratified by TWA-PaO2 on Day 1; Table S2: Baseline characteristics and outcomes of patients stratified by TWA-FiO2 on Day 1; Table S3: Effect of time-varying TWA-PaO2 and TWA-FiO2 on 28-day mortality of 7784 patients with mechanical ventilation; Table S4: Effect of time spent with hyperoxemia and high FiO2 on 28-day mortality of 7784 patients with mechanical ventilation; Table S5: Effect of cumulative exposure of hyperoxemia and high FiO2 on 28-day mortality of 7784 patients with mechanical ventilation; Table S6: Effect of time-varying hyperoxemia and high FiO2 on 28-day mortality after excluding missing data of daily TWA-PaO2 or TWA-FiO2; Table S7: Effect of time-varying hyperoxemia and high FiO2 on 28-day mortality in patients with mechanical ventilation, patients were followed up from inclusion until death, liberated from mechanical ventilation, ICU discharge, or 28 days in the ICU, whichever occurred first; Figure S1: Study flow diagram in present study. Figure S2: Time-varying effect of TWA-PaO2 (A) or TWA-FiO2 (B) on 28-day mortality [file 13054_2022_4114_MOESM2_ESM.docx]
